# Green Tea Polyphenol (–)-Epigallocatechin-3-gallate Protects Endothelial Barrier Function via Myosin Phosphatase and Rho-Kinase

**DOI:** 10.3390/ijms27125166

**Published:** 2026-06-07

**Authors:** Rio Wakasugi, Ayana Shiraki, Ryohei Mitsui, Suguru Nishida, Aya Nishizaki, Shiho Shibata, Rina Fukuda, Kenji Suzuki, Takako Kaneko-Kawano

**Affiliations:** 1 Graduate School of Pharmacy, Ritsumeikan University, 1-1-1 Noji Higashi, Kusatsu, Shiga 525-8577, Japan; 2 College of Pharmaceutical Sciences, Ritsumeikan University, 1-1-1 Noji Higashi, Kusatsu, Shiga 525-8577, Japan

**Keywords:** endothelial cells, EGCG, Rho-kinase, myosin phosphatase, VE-cadherin

## Abstract

Vascular endothelial cells form a selective barrier that regulates the passage of substances and leukocytes between the bloodstream and surrounding tissues, thereby maintaining vascular homeostasis. Although endothelial barrier dysfunction is implicated in numerous diseases, the molecular mechanisms that protect against such dysfunction remain incompletely defined. Thrombin, an inflammatory mediator, increases endothelial permeability by inducing myosin light chain (MLC) phosphorylation through Rho/Rho-associated kinase (Rho-kinase)-mediated inhibition of myosin phosphatase. This process disrupts vascular endothelial cadherin (VE-cadherin)-based junctions and promotes radial stress fiber formation. Here, we demonstrate that the green tea catechin (–)-epigallocatechin-3-gallate (EGCG) reduces phosphorylation of the myosin phosphatase regulatory subunit MYPT1 at inhibitory sites and suppresses Rho-kinase signaling in endothelial cells. Together, these EGCG-mediated effects reduce MLC phosphorylation, inhibit radial stress fiber formation, and preserve VE-cadherin-mediated cell–cell adhesion, thereby maintaining endothelial barrier integrity.

## 1. Introduction

Vascular endothelial cells form a monolayer lining the inner surface of blood vessels, serving as a selective barrier that regulates the exchange of substances and leukocytes between the bloodstream and surrounding tissues [1,2]. Under physiological conditions, this barrier prevents the leakage of plasma components into tissues, whereas inflammatory stimuli transiently increase vascular permeability, allowing proteins and leukocytes to extravasate [3]. Therefore, the vascular endothelial barrier is essential for homeostasis, and its dysfunction contributes to various diseases characterized by excessive vascular permeability, including acute respiratory distress syndrome, asthma, arthritis, ulcerative colitis, and other chronic inflammation [2,4].

Endothelial barrier integrity is primarily maintained by intercellular junctions. Vascular endothelial cadherin (VE-cadherin)-based adherens junctions play a central role in regulating cell–cell adhesion and vascular permeability [5]. VE-cadherin mediates Ca^2+^-dependent homophilic interactions between adjacent endothelial cells [6]. The intracellular domain of VE-cadherin connects to the actin cytoskeleton through adaptor proteins such as α-, β-, and γ-catenin (plakoglobin) [5,7]. When endothelial junctions are stable, cortical actin filaments are organized in parallel beneath the plasma membrane, and VE-cadherin–catenin complexes anchor these filaments to maintain intercellular adhesion [8]. In contrast, when cell–cell adhesions weaken and endothelial permeability increases, actin reorganizes into radial stress fibers that exert contractile forces toward VE-cadherin-based junctions, thereby disrupting the endothelial barrier [8].

Thrombin, an inflammatory mediator, disrupts endothelial cell–cell junctions and increases vascular permeability by inducing the formation of radial stress fibers, which weakens VE-cadherin-mediated adhesion [9]. This cytoskeletal reorganization is mediated by the small GTPase Rho and its effector, Rho-associated kinase (Rho-kinase), which enhances actomyosin contraction by phosphorylating myosin light chain (MLC) [9,10,11]. Rho-kinase promotes MLC phosphorylation through two mechanisms: directly phosphorylating MLC and suppressing myosin phosphatase activity. Rho-kinase directly phosphorylates MLC at threonine 18 (Thr18) and serine 19 (Ser19) to induce actomyosin contraction [12]. Myosin phosphatase is a trimeric enzyme composed of a regulatory subunit (myosin phosphatase targeting subunit 1; MYPT1), a catalytic subunit (protein phosphatase 1 catalytic subunit; PP1c), and a 20 kDa subunit (M20) [13,14,15]. Rho-kinase phosphorylates MYPT1 at Thr696 and Thr853, thereby inhibiting myosin phosphatase activity [16,17,18,19,20,21]. Consequently, thrombin enhances endothelial barrier disruption through dual mechanisms downstream of Rho-kinase: direct phosphorylation of MLC and inhibition of myosin phosphatase activity, both of which promote actomyosin contraction and junctional destabilization.

Tea (*Camellia sinensis*) is a widely consumed beverage worldwide. Catechins are abundant in unfermented green tea, among which (–)-epigallocatechin-3-gallate (EGCG) is the predominant polyphenol in green tea and has been suggested to exert beneficial effects on vascular health [22]. However, the molecular mechanisms underlying these effects remain poorly defined.

EGCG has been reported to activate myosin phosphatase through protein phosphatase 2A (PP2A)-mediated dephosphorylation of MYPT1 at Thr696 in cancer cell lines [23]. EGCG also suppresses endothelial hyperpermeability induced by phorbol 12-myristate 13-acetate (PMA), an artificial activator of protein kinase C (PKC), via PP2A-mediated dephosphorylation of MYPT1 at Thr696 [24]. EGCG has additionally been suggested to inhibit Rho; however, this effect has been observed primarily at concentrations far exceeding those detected in plasma after oral ingestion of green tea or supplements in hepatic stellate cells [25]. Therefore, it remains unclear whether EGCG can protect the integrity of the endothelial barrier disrupted by physiologically relevant inflammatory mediators, such as thrombin, through regulating myosin phosphatase and Rho/Rho-kinase signaling.

In this study, we investigated whether EGCG at non-supraphysiological concentrations can protect endothelial barrier integrity in response to thrombin. We found that EGCG reduces MYPT1 phosphorylation at Thr696 and Thr853 and suppresses Rho-kinase signaling, accompanied by reduced MLC phosphorylation in thrombin-stimulated endothelial cells. EGCG also suppresses thrombin-induced loss of endothelial cell–cell adhesion and radial stress fiber formation, prevents endothelial hyperpermeability, and maintains endothelial barrier integrity. Collectively, these findings suggest that EGCG protects the endothelial barrier from thrombin-induced disruption at non-supraphysiological concentrations.

## 2. Results

### 2.1. EGCG Attenuates Phosphorylation of MYPT1, MLC, and Rho-Kinase

EGCG has been reported to induce the dephosphorylation of MYPT1 at Thr696 in HeLa cells and bovine pulmonary artery endothelial cells [24,26]. However, whether EGCG suppresses MYPT1 phosphorylation at Thr853 remains unclear. Additionally, oral intake of 375–1200 mg of EGCG results in plasma concentrations of approximately 0.6 to 7.4 µM [27,28]. Although EGCG at 100 µM has been reported to suppress Rho activation in hepatic stellate cells [25], this concentration is over 10-fold higher than plasma concentrations achievable after oral intake. Such supraphysiological concentrations may lead to nonspecific effects, complicating the interpretation of the underlying molecular mechanisms. Therefore, we examined whether EGCG at a non-supraphysiological concentration (5 µM) regulates MYPT1 phosphorylation at Thr696 and Thr853 and modulates Rho-kinase signaling in endothelial cells. Thrombin was used at 0.25 U/mL, a concentration previously reported to induce Rho activation and phosphorylation of MYPT1 and MLC in human umbilical vein endothelial cells (HUVECs) [29].

**Figure 1:**
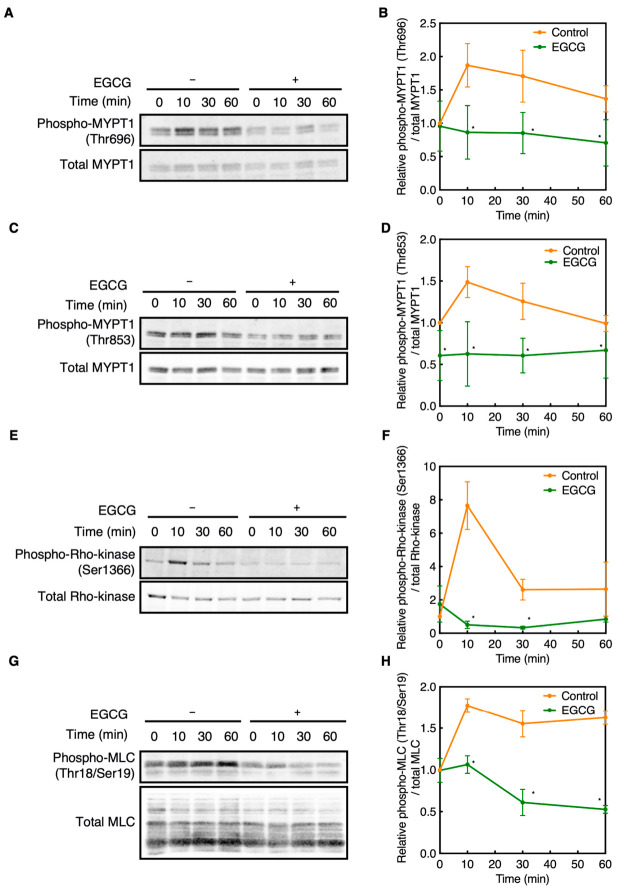
EGCG attenuates the phosphorylation of MYPT1, MLC, and Rho-kinase. Human umbilical vein endothelial cells (HUVECs) were treated with EGCG followed by thrombin for 0, 10, 30, and 60 min. (**A**,**C**,**E**,**G**) Phosphorylated MYPT1 (Thr696 and Thr853), Rho-kinase (Ser1366), MLC (Thr18/Ser19), and the expression levels of MYPT1, Rho-kinase, and MLC were detected by immunoblot analysis. (**B**,**D**,**F**,**H**) The relative levels of phosphorylated MYPT1 (Thr696 and Thr853), Rho-kinase (Ser1366), and MLC (Thr18/Ser19) were quantified from panels (**A**,**C**,**E**,**G**) respectively. Mean ± s.d. (n = 3). Data were analyzed by two-way ANOVA followed by Šídák’s multiple comparisons test. * *p* < 0.05 vs. the corresponding control.

First, we investigated whether 5 µM EGCG regulates MYPT1 phosphorylation in endothelial cells. We induced Rho/Rho-kinase activity with thrombin and assessed phosphorylation of MYPT1 at Thr696 and Thr853 via immunoblotting using anti-phosphorylated Thr696 and Thr853 MYPT1 antibodies. HUVECs were treated with EGCG and stimulated with thrombin for 0–60 min. Thrombin stimulation enhanced MYPT1 phosphorylation at Thr696, whereas EGCG pretreatment attenuated thrombin-induced Thr696 phosphorylation of MYPT1 (Figure 1A,B). Similarly, EGCG pretreatment suppressed thrombin-induced phosphorylation of MYPT1 at Thr853 (Figure 1C,D). These findings indicate that EGCG attenuates MYPT1 phosphorylation at both Thr696 and Thr853 in endothelial cells.

As Rho-kinase is the primary kinase responsible for phosphorylating MYPT1 at Thr853, our results suggest that EGCG at non-supraphysiological concentrations may attenuate Rho-kinase signaling. Therefore, we examined whether 5 µM EGCG attenuates thrombin-induced Rho-kinase signaling. HUVECs were preincubated with or without EGCG, followed by stimulation with thrombin to activate the Rho/Rho-kinase signaling pathway. Activated Rho-kinase undergoes autophosphorylation at Ser1366 [30]. To assess Rho-kinase activity, we measured its phosphorylation status at Ser1366. Thrombin stimulation increased Rho-kinase phosphorylation at Ser1366 (Figure 1E,F). In contrast, EGCG pretreatment attenuated Rho-kinase phosphorylation after thrombin stimulation (Figure 1E,F). These results suggest that EGCG at non-supraphysiological concentrations also attenuates Rho-kinase signaling in vascular endothelial cells.

Finally, we investigated whether EGCG regulates MLC phosphorylation in endothelial cells. HUVECs were pretreated with EGCG, followed by stimulation with thrombin. Thrombin stimulation induced MLC phosphorylation at Thr18/Ser19, whereas EGCG attenuated this phosphorylation (Figure 1G,H). These results suggest that EGCG at a non-supraphysiological concentration attenuates MLC phosphorylation at Thr18/Ser19, possibly by reducing MYPT1 phosphorylation at Thr696 and Thr853 and by modulating Rho-kinase signaling.

### 2.2. EGCG Maintains Vascular Endothelial Cell Adhesion

**Figure 2:**
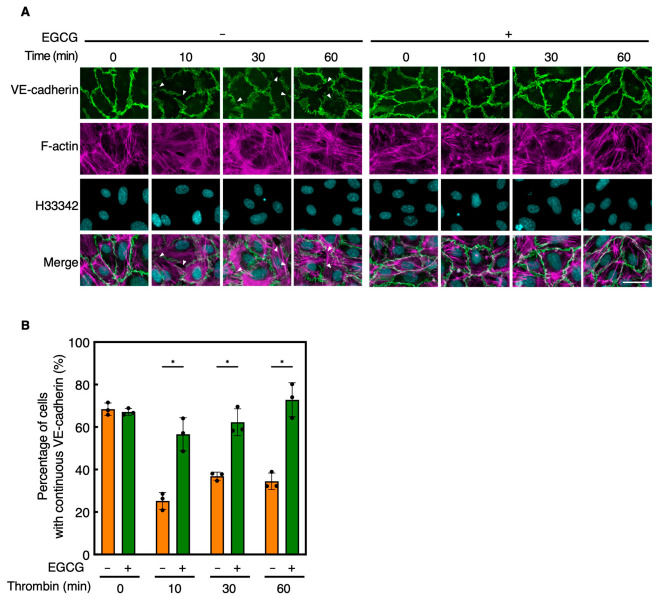
EGCG maintains vascular endothelial cell adhesion. (**A**) HUVECs were stimulated with thrombin after preincubation with EGCG. Localization of VE-cadherin, F-actin (rhodamine–phalloidin), and nuclei (Hoechst 33342) was detected using immunostaining. Scale bar, 50 μm. Arrowheads indicate sites of VE-cadherin regression and intercellular gaps. The original uncropped full-field images are provided in Supplementary Figure S2. (**B**) The percentage of HUVECs exhibiting continuous VE-cadherin around the entire cell periphery was quantified. Mean ± s.d. (n = 3). Student’s *t*-test vs. -EGCG condition, * *p* < 0.05.

When cell–cell adhesion is stable, the actin cytoskeleton is organized as parallel cortical actin beneath the plasma membrane. In contrast, contraction of radial actin stress fibers destabilizes endothelial cell–cell adhesion and increases vascular permeability [8]. Thrombin enhances endothelial permeability by inducing stress fiber formation and actomyosin contraction via the Rho/Rho-kinase signaling pathway [9]. Our findings suggest that EGCG suppresses thrombin-induced upregulation of Rho-kinase signaling (Figure 1A–H). Therefore, we examined whether EGCG suppresses thrombin-induced stress fiber formation and the disruption of cell–cell adhesion in HUVECs. After incubation with or without EGCG, HUVECs were treated with thrombin for 0–60 min. Without thrombin or EGCG, VE-cadherin localized at cell–cell junctions in HUVECs (Figure 2A). In contrast, thrombin treatment caused jagged and discontinuous VE-cadherin staining in a time-dependent manner (Figure 2A, arrowheads). However, EGCG attenuated this thrombin-induced disruption of VE-cadherin localization (Figure 2A). Moreover, although thrombin induced stress fiber formation in a time-dependent manner, EGCG suppressed this response and preserved cortical actin (Figure 2A).

To evaluate the continuity of endothelial cell–cell adhesion, we also quantified the percentage of HUVECs exhibiting continuous VE-cadherin staining along the cell periphery. In non-stimulated cells, 68% showed continuous VE-cadherin at cell–cell junctions. Thrombin stimulation markedly reduced this proportion, with only 25%, 37%, and 35% of HUVECs maintaining continuous junctional VE-cadherin after 10, 30, and 60 min of thrombin treatment, respectively (Figure 2B, orange bars). In contrast, EGCG preincubation suppressed the thrombin-induced loss of continuous VE-cadherin localization. Under EGCG preincubation, 67%, 57%, 62%, and 73% of HUVECs maintained continuous junctional VE-cadherin after 0, 10, 30, and 60 min of thrombin stimulation, respectively (Figure 2B, green bars). Because 18 h of serum starvation and EGCG treatment increased the tendency toward partial endothelial cell detachment, the immunofluorescence experiments shown in Figure 2 were performed after 6 h of serum starvation and EGCG pretreatment. However, similar effects of EGCG on VE-cadherin localization and stress fiber formation were also observed after 18 h of EGCG pretreatment and serum starvation (Supplementary Figure S1). In addition, we quantified non-junctional F-actin fluorescence intensity, excluding cortical actin associated with cell–cell junctions, as an indicator of stress fiber formation. Thrombin stimulation increased non-junctional F-actin fluorescence intensity, whereas EGCG suppressed this increase (Supplementary Figure S3). These results suggest that EGCG preserves endothelial cell–cell adhesion and suppresses radial stress fiber formation.

### 2.3. EGCG Protects Vascular Endothelial Barrier Function

**Figure 3:**
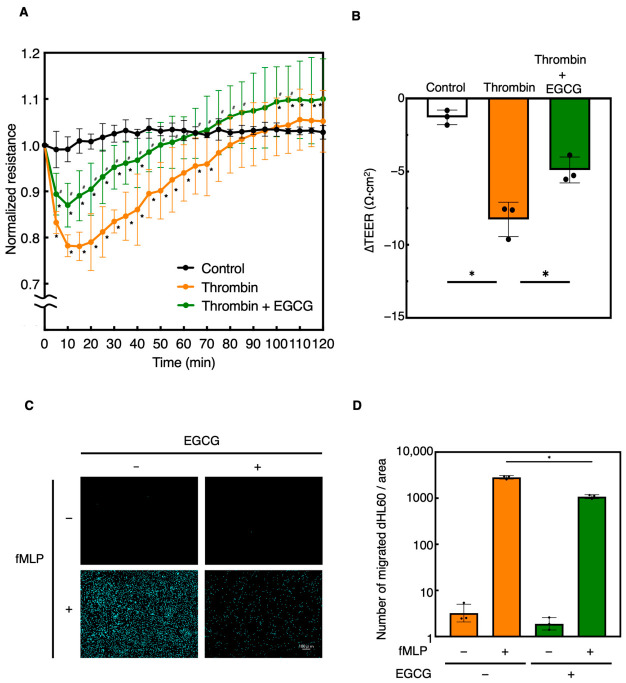
EGCG protects vascular endothelial barrier function. (**A**) HUVECs were treated with EGCG, followed by thrombin. Endothelial permeability was sequentially measured by transendothelial electrical resistance (TEER). Mean ± s.d. (n = 3). Data were analyzed by two-way repeated measures ANOVA followed by Tukey’s multiple comparisons test. * *p* < 0.05 vs. Control; ^#^ *p* < 0.05 vs. Thrombin. (**B**) ΔTEER (Ω·cm^2^) was calculated as the minimum TEER value minus the starting TEER value (0 min). Mean ± s.d. (n = 3). Student’s *t*-test (Control vs. Thrombin, Thrombin vs. Thrombin + EGCG), * *p* < 0.05. (**C**) HUVEC monolayers were cultured on the upper chamber (insert) of the Boyden chamber and preincubated with EGCG. Differentiated human promyelocytic leukemia cells (dHL-60) were added onto the HUVEC monolayer on the insert. Formyl-methionyl-leucyl-phenylalanine (fMLP) was added into the bottom chamber to induce dHL-60 transmigration. The nuclei stained with Hoechst 33342 on the underside of the membrane facing the bottom chamber were detected as transmigrated dHL-60. Scale bar, 100 μm. (**D**) The number of transmigrated dHL-60 cells was quantified. For each condition, nuclei were counted in five microscopic fields per well, and three wells were analyzed. The mean value from all fields and wells was calculated for each independent experiment. Experiments were repeated independently three times. Mean ± s.d. (n = 3). Student’s *t*-test vs. the −EGCG + fMLP condition, * *p* < 0.05. Each dot represents an independent experiment.

To examine whether EGCG protects endothelial barrier function, we measured temporal changes in endothelial permeability using transendothelial electrical resistance (TEER). HUVECs were cultured on transwell inserts until a monolayer had formed. The monolayers were then preincubated with or without EGCG, followed by thrombin stimulation. Resting HUVEC monolayers exhibited constant TEER values, indicating low endothelial permeability and an intact endothelial barrier function (Figure 3A, black line). In contrast, thrombin decreased TEER in HUVEC monolayers, reaching a minimum at 10–15 min and returning toward baseline by 75 min after stimulation (Figure 3A; orange line). EGCG preincubation attenuated the thrombin-induced reduction in TEER and accelerated recovery, restoring TEER to baseline levels by 45 min (Figure 3A, green line). We also calculated the difference between the minimum and starting values (ΔTEER) (Figure 3B). These results indicate that EGCG exerts a protective effect against thrombin-induced disruption of the endothelial barrier.

Finally, to examine whether EGCG protects endothelial barrier function against neutrophil migration through vascular endothelial cells (transmigration), we performed a Boyden chamber assay using differentiated human promyelocytic leukemia cells (dHL-60 cells) as a neutrophil model. HUVECs were cultured on the membrane of the Boyden chamber insert until a confluent monolayer formed. The monolayers were then preincubated with or without EGCG. Meanwhile, the nuclei of dHL-60 cells were stained with Hoechst 33342 and seeded onto the HUVEC monolayers. Formyl-methionyl-leucyl-phenylalanine (fMLP), a peptide that induces neutrophil migration, was added at the bottom chamber to stimulate transmigration. All transmigration assays were performed under basal conditions in the absence of thrombin stimulation. After 60 min, dHL-60 cell nuclei were observed on the membrane surface facing the bottom chamber, representing cells that had transmigrated through the HUVEC monolayer. Although few dHL-60 cells transmigrated in the absence of fMLP, fMLP stimulation markedly increased the number of nuclei visible on the underside of the membrane (Figure 3C). In contrast, EGCG preincubation of HUVEC monolayers reduced fMLP-induced dHL-60 transmigration (Figure 3C). We next quantified transmigration by counting dHL-60 nuclei on the underside of the membrane. Consistent with the microscopy results (Figure 3C), fMLP stimulation substantially increased the number of transmigrated dHL-60 cells, whereas EGCG preincubation significantly reduced this number (Figure 3D). These results suggest that EGCG preserves the endothelial barrier and suppresses neutrophil transmigration across endothelial monolayers.

## 3. Discussion

**Figure 4:**
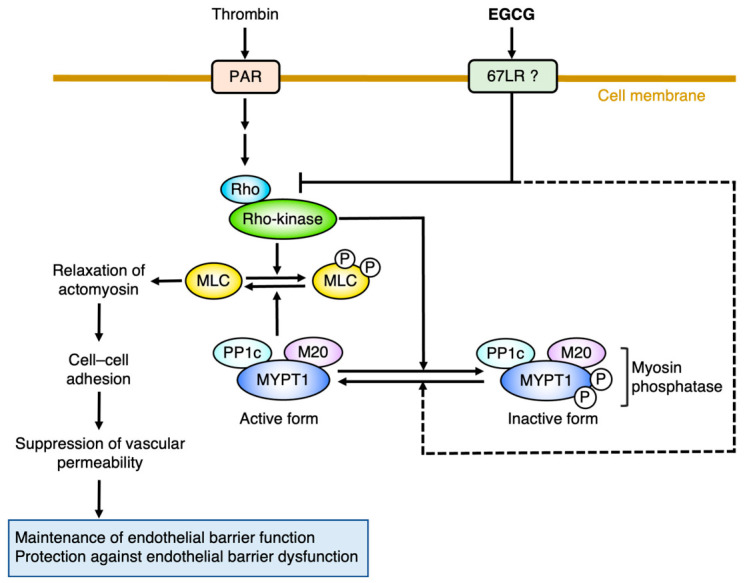
EGCG protects the vascular endothelial barrier. Inflammatory mediators (e.g., thrombin) activate the Rho/Rho-kinase signaling pathway, enhancing actomyosin contraction and stress fiber formation through MLC phosphorylation. Rho-kinase directly phosphorylates MLC and suppresses myosin phosphatase through MYPT1 phosphorylation. Actomyosin contraction attenuates endothelial cell–cell adhesion, leading to vascular permeability. Our findings suggest that EGCG suppresses thrombin-induced upregulation of Rho-kinase signaling, accompanied by reduced phosphorylation of MYPT1 and MLC, thereby helping maintain endothelial barrier function. It has also been reported that EGCG enhances myosin phosphatase activity through the PKA/PP2A signaling pathway [24], which cannot be excluded as a contributing mechanism (dashed line).

In this study, we found that EGCG attenuates thrombin-induced MYPT1 phosphorylation at Thr696 and Thr853, and MLC phosphorylation at Thr18/Ser19. We also found that EGCG protects vascular endothelial cells against thrombin-induced disruption of cell–cell adhesion and suppresses radial stress fiber formation. Consequently, EGCG preserves endothelial barrier integrity by maintaining cell–cell adhesion and suppressing thrombin-induced hyperpermeability (Figure 4). Furthermore, EGCG decreases neutrophil transmigration by enhancing endothelial barrier function. Importantly, all these effects were observed at non-supraphysiological concentrations of EGCG. Therefore, our findings suggest that EGCG protects against endothelial barrier dysfunction induced by inflammatory mediators such as thrombin. These protective effects may involve suppression of thrombin-induced upregulation of Rho-kinase signaling, accompanied by reduced phosphorylation of MYPT1 and MLC.

Several studies have shown that EGCG attenuates endothelial hyperpermeability. EGCG has been shown to suppress angiotensin II-induced increases in vascular permeability, an effect accompanied by inhibition of the p38 MAPK pathway in HUVECs [31]. In addition, EGCG has been reported to attenuate endothelial hyperpermeability induced by inflammatory stimuli, such as TNF-α or lipopolysaccharide, and to reduce NF-κB-dependent inflammatory gene expression in endothelial cells [32,33]. EGCG has also been reported to suppress VEGF-induced retinal vascular hyperpermeability by regulating angiogenic factors in vivo [34]. Furthermore, under PMA stimulation, an artificial activator of PKC, EGCG has been reported to suppress the increase in MYPT1 phosphorylation at Thr696 and to attenuate endothelial permeability [24]. Collectively, these studies indicate that EGCG suppresses barrier dysfunction induced by artificial stimuli or transcription-dependent inflammatory pathways. However, it remains unclear whether EGCG suppresses inflammatory mediator-induced endothelial hyperpermeability by regulating myosin phosphatase and Rho/Rho-kinase signaling. Our findings suggest that EGCG may suppress acute endothelial barrier disruption induced by inflammatory mediators through suppression of Rho-kinase signaling, accompanied by reduced MYPT1 and MLC phosphorylation, rather than through transcription-dependent mechanisms.

Thrombin activates Rho, leading to Rho-kinase activation [9,10,11]. Activated Rho-kinase phosphorylates MYPT1 at Thr696 and Thr853, thereby inhibiting myosin phosphatase activity, and directly phosphorylates MLC at Thr18/Ser19, resulting in enhanced MLC phosphorylation [9,11]. This increase in MLC phosphorylation promotes actomyosin contraction and the formation of radial stress fibers, thereby weakening endothelial cell–cell adhesion and subsequently increasing vascular endothelial permeability in response to thrombin [9,11,35].

EGCG enhances myosin phosphatase activity through a protein kinase A (PKA)–PP2A–MYPT1 signaling cascade [24]. The 67 kDa laminin receptor (67LR) has been identified as a receptor for EGCG, and its activation triggers PKA signaling [23,36]. PKA-dependent activation of PP2A promotes dephosphorylation of MYPT1 at Thr696, thereby increasing myosin phosphatase activity [23,24]. In addition, PKA is known to inhibit Rho activity [37]. Therefore, EGCG may suppress the Rho/Rho-kinase signaling pathway through PKA activation. Indeed, extremely high concentrations of EGCG (100 µM) suppress Rho activation in hepatic stellate cells [25]. However, given that these concentrations far exceed the plasma levels achievable in humans via oral intake, it remains unclear whether EGCG at lower, non-supraphysiological concentrations suppresses Rho/Rho-kinase signaling in endothelial cells. Oral EGCG doses of 375–1200 mg/day result in maximum plasma concentrations of approximately 0.6–7.4 µM [27,28]. In general, when small-molecule compounds are used at concentrations far exceeding those achievable, they are more likely to exert nonspecific effects on molecules other than their intended targets. Under such conditions, it becomes difficult to clearly identify the molecular mechanisms underlying the observed effects. Therefore, in this study, we focused on EGCG at a non-supraphysiological concentration and analyzed the signaling pathways associated with endothelial barrier function.

In this study, we found that 5 µM EGCG attenuates phosphorylation of Rho-kinase at Ser1366, a marker of Rho-kinase activation, and reduces phosphorylation of MYPT1 at Thr853, a Rho-kinase-dependent site (Figure 1C–F). These results suggest that EGCG at non-supraphysiological concentrations also attenuates Rho-kinase signaling. Furthermore, at these concentrations, EGCG reduces MLC phosphorylation at Thr18/Ser19 (Figure 1G,H) and inhibits radial stress fiber formation (Figure 2A). Collectively, these findings suggest that EGCG suppresses thrombin-induced upregulation of the Rho-kinase signaling pathway, accompanied by reduced phosphorylation of MYPT1 at Thr696 and Thr853 and reduced MLC phosphorylation at Thr18/19.

The stabilization of cortical actin structures preserves VE-cadherin-mediated cell–cell adhesion and maintains endothelial barrier integrity [8]. Our results indicate that EGCG at non-supraphysiological concentrations suppresses thrombin-induced disruption of VE-cadherin by inhibiting MLC phosphorylation-driven radial stress fiber formation (Figure 2A,B). Through this mechanism, EGCG facilitates the maintenance of endothelial barrier integrity following thrombin stimulation (Figure 3A,B). In addition, we found that EGCG suppresses fMLP-induced neutrophil transmigration across the vascular endothelium by preserving the endothelial barrier under basal conditions (Figure 3C,D). These results suggest that EGCG may contribute to the maintenance of endothelial barrier function and thereby limit abnormal leakage of plasma components and leukocytes from the bloodstream into tissues.

Our findings suggest that EGCG at non-supraphysiological concentrations suppresses inflammatory mediator-induced endothelial hyperpermeability and maintains endothelial barrier integrity, accompanied by reduced Rho-kinase signaling and decreased inhibitory phosphorylation of MYPT1. Therefore, EGCG-mediated preservation of endothelial integrity may provide mechanistic insight into pathological conditions associated with excessive vascular permeability, in a preventive context. In this study, collagen coating was applied under identical conditions for all experiments. However, different substrates (plastic, glass, and Transwell filters) may vary in stiffness and surface architecture; therefore, their potential effects on cytoskeletal organization, actomyosin contractility, stress fiber formation, and cell–cell junction organization cannot be ruled out. The influence of substrate-dependent mechanical factors should be considered when interpreting the findings. Furthermore, all experiments were performed using HUVECs. Therefore, whether the findings obtained in this study can be generalized to other endothelial cell types requires further investigation.

## 4. Materials and Methods

### 4.1. Cell Cultures

Human umbilical vein endothelial cells (HUVECs; KE-4109) and human promyelocytic leukemia cells (HL-60; JCRB0085) were obtained from Kurabo Industries Ltd. (Osaka, Japan) and the Japanese Collection of Research Bioresources (JCRB) Cell Bank (Osaka, Japan), respectively. HUVECs and HL-60 were cultured at 37 °C in a humidified atmosphere containing 5% CO_2_.

HUVECs were grown in Humedia-EB2 medium (KE-2350S, Kurabo, Osaka, Japan) containing 2% (*v*/*v*) fetal bovine serum (FBS) and a growth supplement mixture (10 ng/mL human epidermal growth factor, 1.34 µg/mL hydrocortisone hemisuccinate, 50 µg/mL gentamicin, 50 ng/mL amphotericin B, 5 ng/mL human basic fibroblast growth factor, and 10 µg/mL heparin) (KE-6150, Kurabo). The culture medium was refreshed every other day. For the experiments described below, HUVECs at passage 6 were seeded onto culture dishes, glass coverslips, or transwell inserts coated with collagen (10 µg/mL collagen (PSC-1-201-20, Nippi, Tokyo, Japan) dissolved in 5 mM acetic acid (017-00256, Fujifilm Wako, 017-00256).

HL-60 cells were cultured in RPMI-1640 (189-02025, Fujifilm Wako, Osaka, Japan) supplemented with 20% FBS (A5256701, Thermo Fisher Scientific, Waltham, MA, USA). To induce differentiation into neutrophil-like cells, HL-60 cells were incubated in RPMI-1640 containing 1.3% DMSO (13445-74, Nacalai Tesque, Kyoto, Japan) and 10% FBS for 5–7 d.

### 4.2. Immunoblot Analysis of Phosphorylated MYPT1, MLC, and Rho-Kinase

HUVECs (2.5 × 10^5^ cells) were seeded onto collagen-coated 3.5 cm dishes and cultured for 24 h. The culture medium was replaced with FBS- and growth supplement-free Humedia-EB2 containing 5 µM EGCG (E4143, Sigma-Aldrich, St Louis, MO, USA; stock solution prepared in sterile water), and the cells were incubated for 18 h. The medium was then replaced with medium containing 5 µM EGCG and 0.25 U/mL thrombin (206-18411, Fujifilm Wako), and cells were incubated for 0–60 min.

Cells were collected and precipitated with trichloroacetic acid (TCA) for immunoblot analysis, as described below. Cells were washed twice with phosphate-buffered saline (PBS) and collected using 10% (*w*/*v*) TCA (204-16212, Fujifilm Wako) supplemented with 2 mM dithiothreitol (DTT; 14112-94, Nacalai Tesque) on ice. Collected cell samples were centrifuged at 20,400× *g* for 15 min at 4 °C. Pellets were washed three times with ice-cold acetone containing 2 mM DTT and solubilized in sample buffer (0.19 M Tris-HCl (pH 6.8), 3% sodium dodecyl sulfate (SDS), 2% 2-mercaptoethanol, 1.1% glycerol, and bromophenol blue) by rotating at room temperature for 1 h. These solubilized samples were incubated at 95 °C for 10 min and were subsequently used for immunoblotting analysis.

The proteins of TCA-precipitated samples were separated by SDS-PAGE and transferred onto polyvinylidene difluoride (PVDF) membranes (10600021, Cytiva, Marlborough, MA, USA). Immunoblotting was performed using the following primary and secondary antibodies. Primary antibodies were anti-phospho-Thr696 MYPT1 (1:500 dilution; ABS45, Millipore, Burlington, MA, USA), anti-phospho-Thr853 MYPT1 (1:1000 dilution; 36-003, Millipore), anti-MYPT1 (1:500 dilution; 612164, BD Biosciences, San Jose, CA, USA), anti-phospho-Ser1366 ROCK2 (1:250 dilution; MA5-42377, Invitrogen, Waltham, MA, USA), anti-ROCK2 (1:50 dilution; sc-398519, Santa Cruz Biotechnology, Dallas, TX, USA), anti-phospho-Thr18/Ser19 MLC (1:100 dilution; #3674, Cell Signaling Technology, Danvers, MA, USA), and anti-MLC (1:200 dilution; M4401, Sigma-Aldrich). Secondary antibodies were IRDye 680CW goat anti-rabbit IgG (1:1000; 926-68071, LI-COR, Lincoln, NE, USA) or IRDye 800CW goat anti-mouse IgG (1:1000; 926-32210, LI-COR). Signals were visualized with the Odyssey Fc imaging system (LI-COR) and quantified using Image Studio software version 2.0 (LI-COR). For quantification, phosphorylated protein levels were normalized to the corresponding total protein levels detected on the same membrane. The values were further normalized to the control condition (0 min, without EGCG), which was set to 1.0.

### 4.3. Immunostaining

HUVECs (1.5 × 10^5^ cells) were seeded onto collagen-coated glass coverslips (C013001, Matsunami, Osaka, Japan) in 24-well culture plates and cultured for 72 h, with the medium changed every day. Subsequently, HUVECs were treated with 5 µM EGCG in Humedia-EB2 without FBS or growth supplements for 6 h (18 h in Supplementary Figure S1). The culture medium was then replaced with medium containing 5 µM EGCG and 0.25 U/mL thrombin, and cells were incubated for 0–60 min. At each time point, the HUVECs were washed with PBS and fixed with 3.7% formaldehyde (16223-55, Nacalai Tesque) in PBS for 10 min. Fixed HUVECs were permeabilized with 0.2% Triton X-100 (35501-15, Nacalai Tesque) and 0.2% bovine serum albumin (BSA; 013-15143, Fujifilm Wako) in PBS for 10 min and then blocked with 1% BSA in PBS for 60 min. HUVECs were subsequently incubated with anti-VE-cadherin antibody (1:100 dilution; sc-9989, Santa Cruz Biotechnology) for 60 min at room temperature. Following PBS washes, cells were briefly blocked again with 1% BSA in PBS for 5 min and incubated with Hoechst 33342 (1:4000 dilution; B2261, Sigma-Aldrich), rhodamine–phalloidin (1:50 dilution; PHDR1, Cytoskeleton, Denver, CO, USA), and Alexa Fluor 488 goat anti-mouse IgG (1:300 dilution; A11029, Life Technologies, Waltham, MA, USA) for 60 min at room temperature. Fluorescence images were acquired using a BZ-X710 microscope (Keyence, Osaka, Japan) equipped with a 40× objective lens (Plan Apo λ, Nikon, Tokyo, Japan).

As an indicator of the continuity of endothelial cell–cell adhesion, the percentage of HUVECs exhibiting continuous VE-cadherin staining at cell–cell junctions was automatically quantified using the Hybrid Cell Count Analyzer (BZ-H3G, Keyence). All images were acquired under identical exposure conditions. To ensure objectivity, identical analysis parameters and threshold settings were applied to all images. After background signal removal, regions continuously enclosed by VE-cadherin staining were automatically identified, and the number of nuclei within each region was quantified. When a VE-cadherin-enclosed region contained a single nucleus, VE-cadherin was considered to be continuously localized along the entire cell periphery, indicating intact VE-cadherin-mediated cell–cell adhesion. In contrast, when a single VE-cadherin–positive region contained two or more nuclei, VE-cadherin continuity was considered to be disrupted, resulting in indistinct boundaries between adjacent cells, indicating partial loss of VE-cadherin-mediated cell–cell adhesion. For each independent experiment, one representative image covering a wide central area of the coverslip was acquired and used for quantitative analysis. Although the immunostaining images shown in Figure 2A were cropped for presentation purposes, the original images covering a wide central area of the coverslip were used for quantification. All cells within the capture field were included in the analysis and automatically quantified without manual selection. More than 60 cells were analyzed per image.

Non-junctional F-actin fluorescence intensity was quantified as an indicator of stress fiber formation. To distinguish stress fiber-associated F-actin from cortical actin localized at cell–cell junctions, F-actin signals overlapping with VE-cadherin–positive cell–cell adhesion regions and the surrounding 1 μm area were excluded from the analysis. The remaining cytoplasmic F-actin fluorescence intensity was measured per cell. Cell number was determined by nuclear staining. This parameter was used as an index of stress fiber formation.

Cell viability was not directly assessed in the present study. However, in the 18 h of EGCG pretreatment and serum starvation experiments (Supplementary Figure S1), endothelial monolayers remained largely intact, although a modest effect of prolonged EGCG treatment or serum starvation on cell viability or cell attachment could not be excluded.

### 4.4. Measurement of Transendothelial Electrical Resistance (TEER)

HUVECs (2.3 × 10^4^ cells) were seeded onto collagen-coated polyethylene terephthalate membranes of transwell inserts (0.3 cm^2^, 0.4 µm pore size; 353095, Falcon, Corning, NY, USA) in 24-well culture plates and cultured for 72 h. Cells were incubated with FBS-and growth supplement-free Humedia-EB2 containing 1 µM EGCG for 18 h. TEER was continuously monitored from the start of serum starvation and EGCG treatment using the ECIS TEER24 (Applied Biophysics, Troy, NY, USA), which continuously records TEER values from the same well over time. After 18 h of EGCG treatment, thrombin was added to the culture medium to a final concentration of 0.25 U/mL, and TEER was continuously monitored for 120 min. One well per condition was used in each of the three independent experiments. After thrombin stimulation, the TEER value (Ω·cm^2^) at 0 min was set to 1.0, and the TEER values at each time point were expressed as ratios relative to this baseline value and defined as normalized resistance. ΔTEER (Ω·cm^2^) was calculated as the minimum TEER value minus the starting TEER value (0 min). The TEER of HUVECs prior to thrombin stimulation, after correction with the resistance of cell-free wells, was approximately 40–50 ohm·cm^2^. No apparent difference in TEER was observed between EGCG-pretreated and untreated HUVECs.

### 4.5. Transmigration Assay (Boyden Chamber Assay)

HUVECs (2.3 × 10^4^ cells) were seeded onto 0.3 cm^2^ collagen-coated polycarbonate membranes of transwell inserts (3.0 µm pore size; 3415, Corning, Corning, NY, USA) and cultured for 96 h. Under these conditions, 18 h of serum starvation and EGCG treatment were associated with an increased tendency toward partial detachment of the endothelial monolayer. Therefore, a 2 h EGCG pretreatment period was used in the transmigration experiments. HUVEC monolayers were preincubated with 0 or 5 µM EGCG and serum-starved for 2 h. Nuclei of differentiated HL-60 (dHL-60) were stained with Hoechst 33342 (1:4000 dilution) and seeded onto HUVEC monolayers. dHL-60 transmigration was stimulated by adding 10 nM formyl-methionyl-leucyl-phenylalanine (fMLP; F3506, Sigma-Aldrich) to the bottom chamber in the absence of EGCG. After 60 min of fMLP stimulation, transmigrated dHL-60 cells were identified by stained nuclei on the membrane surface facing the bottom chamber. For each independent experiment, three wells per condition were analyzed, and five images were acquired per well. Nuclei within each field were automatically counted using a Hybrid Cell Count Analyzer (BZ-H3G, Keyence). Experiments were repeated three times independently.

### 4.6. Statistics and Reproducibility

Values are expressed as mean ± standard deviation (s.d.) from three independent experiments. Statistical analyses were performed using Prism 10 (GraphPad). Data were analyzed using Student’s *t*-test, two-way ANOVA followed by Šídák’s multiple comparisons test, and two-way repeated measures ANOVA followed by Tukey’s multiple comparisons test. Statistical significance was defined as *p* < 0.05.

## Data Availability

The datasets used and/or analysed during the current study are available from the corresponding author on reasonable request.

## Supplementary Materials

The following supporting information can be downloaded at https://www.mdpi.com/article/10.3390/ijms27125166/s1.

## Abbreviations

67LR	67 kDa laminin receptor
EGCG	(–)-epigallocatechin-3-gallate
HL-60	human promyelocytic leukemia cells
HUVECs	human umbilical vein endothelial cells
M20	20 kDa subunit
MLC	myosin light chain
MYPT1	myosin phosphatase targeting subunit 1
PKA	protein kinase A
PKC	protein kinase C
PMA	phorbol 12-myristate 13-acetate
PP1c	protein phosphatase 1 catalytic subunit
PP2A	protein phosphatase 2A
Rho-kinase	Rho-associated kinase
VE-cadherin	vascular endothelial cadherin
