# Supplementary material for: Green Tea Polyphenol (–)-Epigallocatechin-3-gallate Protects Endothelial Barrier Function via Myosin Phosphatase and Rho-Kinase
Source: Int J Mol Sci. 2026 Jun 7;27(12):5166. doi: 10.3390/ijms27125166 (PMC13299414; doi:10.3390/ijms27125166)
Supplement: Supplementary file 1 [file ijms-27-05166-s001.zip › ijms-4329375-supplementary.pdf]

Supplementary Materials

Green tea polyphenol (–)-epigallocatechin-3-gallate protects endothelial barrier function via myosin phosphatase and Rho-kinase

Rio Wakasugi[1], Ayana Shiraki[1], Ryohei Mitsui[2], Suguru Nishida[2], Aya Nishizaki[2], Shiho Shibata[2], Rina Fukuda[2], Kenji Suzuki[1, 2], and Takako Kaneko-Kawano[1, 2, *]

[1]Graduate School of Pharmacy, Ritsumeikan University, 1-1-1 Noji Higashi, Kusatsu, Shiga 525-8577, Japan

[2]College of Pharmaceutical Sciences, Ritsumeikan University, 1-1-1 Noji Higashi, Kusatsu, Shiga 525-8577, Japan

*Address for Correspondence: Takako Kaneko-Kawano, Graduate School of Pharmacy, Ritsumeikan University, 1-1-1 Noji Higashi, Kusatsu, Shiga 525-8577, Japan

Telephone: +81-77-561-3907

E-mail: takanek@fc.ritsumei.ac.jp

A

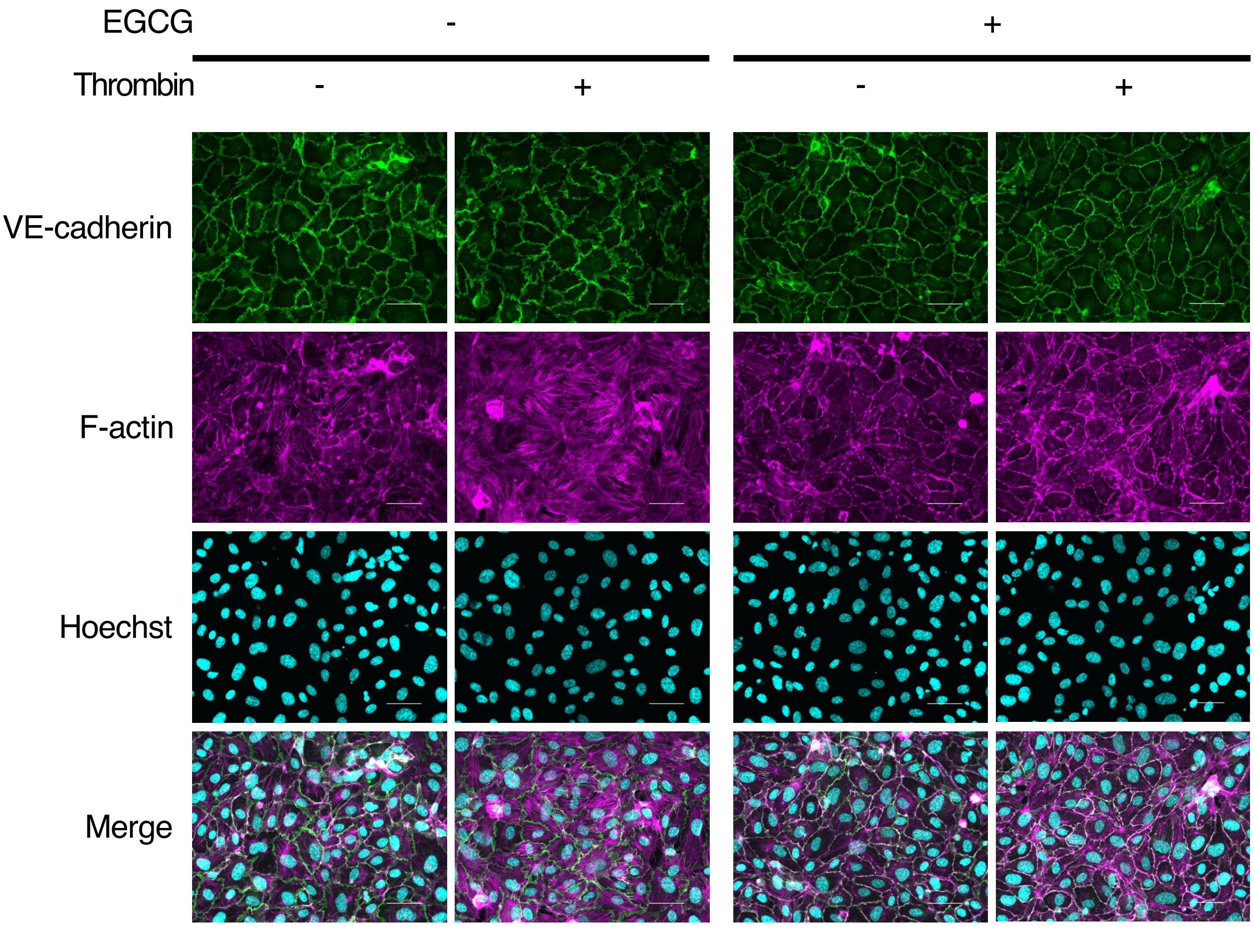

B

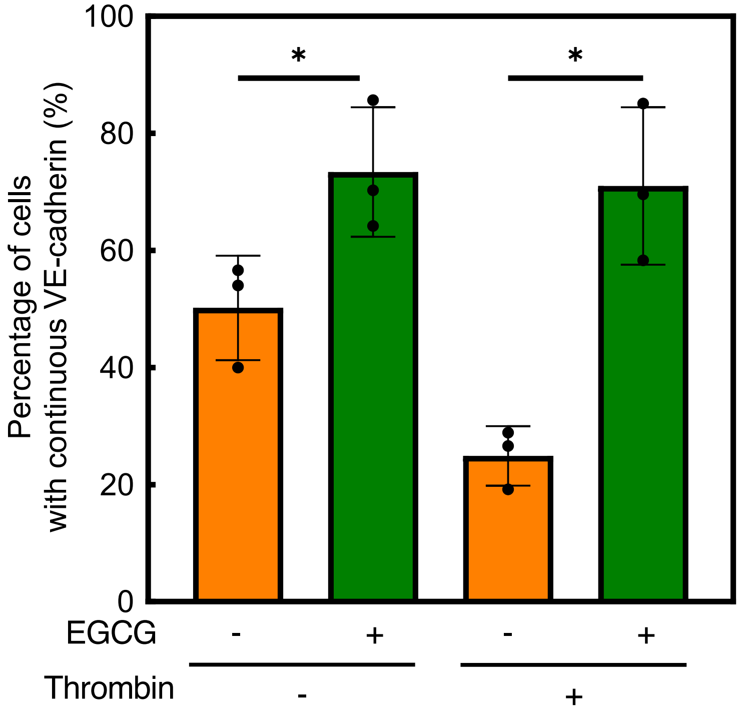

Supplementary Figure S1. EGCG maintains vascular endothelial cell–cell adhesion under 18 h preincubation conditions. **A.** HUVECs were stimulated with 0.25 U/mL thrombin for 60 min after preincubation with 5 μM EGCG for 18 h. Localization of VE-cadherin, F-actin (rhodamine–phalloidin), and nuclei (Hoechst 33342) was detected using immunostaining. Scale bar, 50 μm. **B.** The percentage of HUVECs exhibiting continuous VE-cadherin around the entire cell periphery was quantified. Mean ± s.d. (n = 3). Student's *t*-test vs. –EGCG condition, *$p < 0.05$.

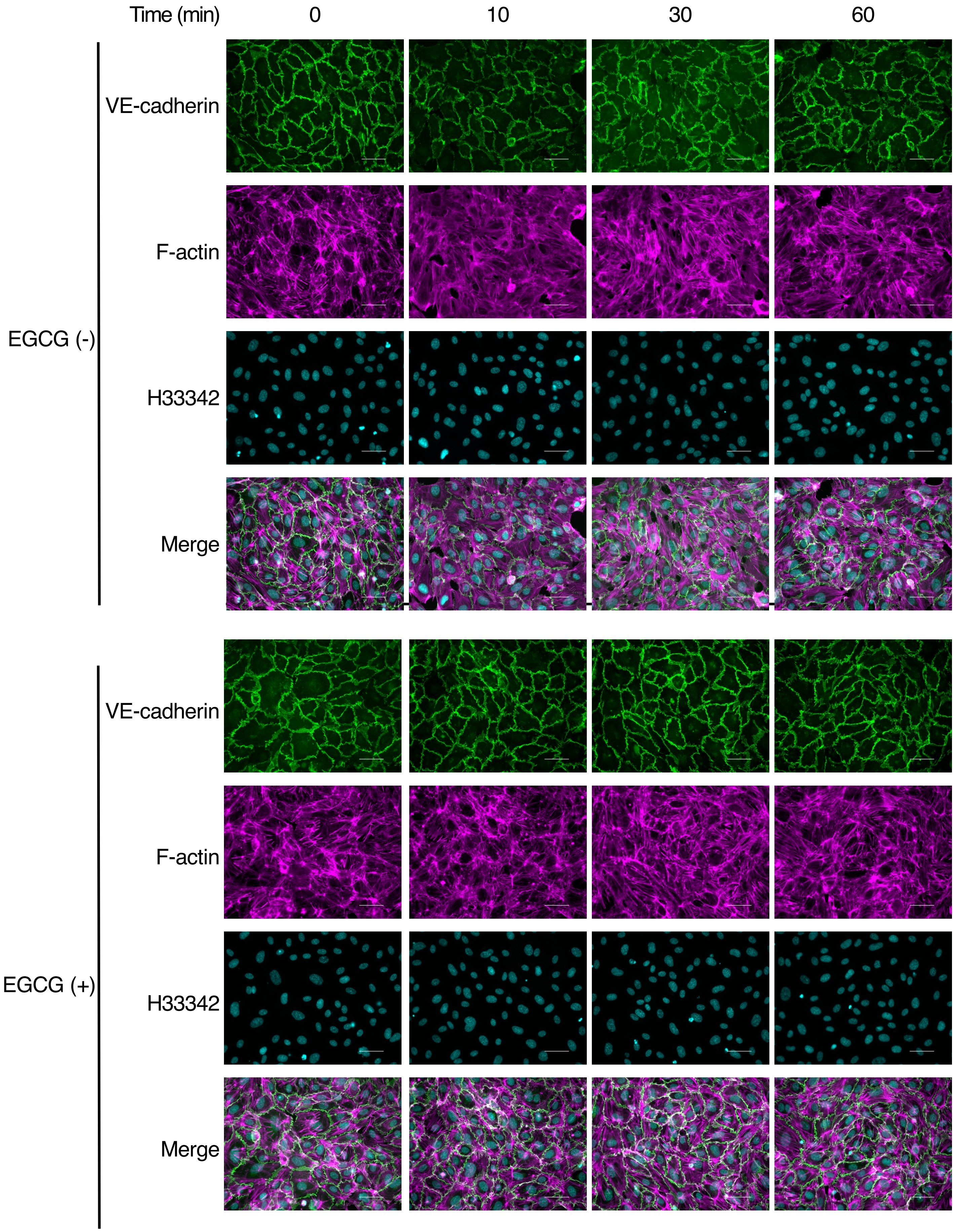

Supplementary Figure S2. Uncropped immunofluorescence images corresponding to Figure 2A. Scale bar, 50 μm.

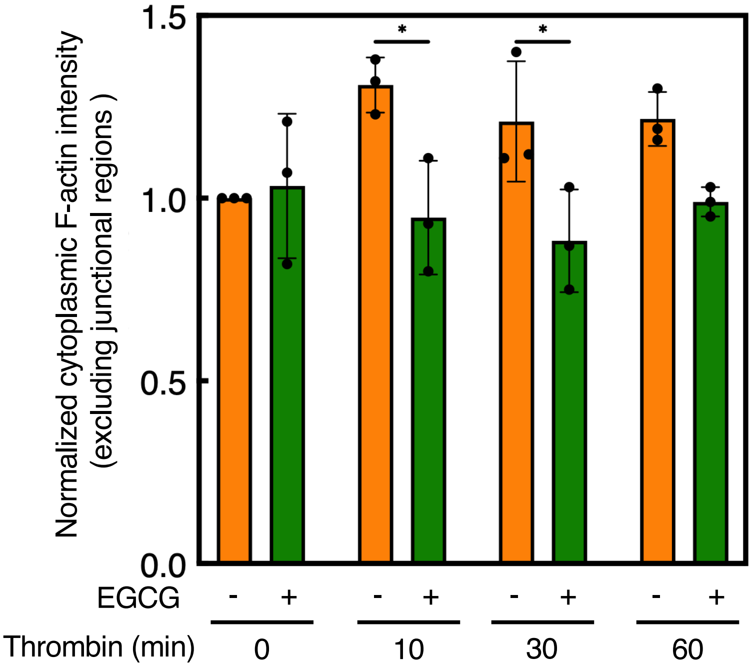

Supplementary Figure S3. F-actin intensity was quantified in cytoplasmic regions excluding VE-cadherin–positive junctional areas to minimize the contribution of cortical actin. Quantification was performed using uncropped full-field images (Supplementary Figure S2). The mean F-actin intensity per cell was measured and normalized to the control condition. Data are presented as mean ± s.d. (n = 3). Student's *t*-test vs. -EGCG condition, *$p < 0.05$.
